# Developmental profiling of ASD-related *shank3* transcripts and their differential regulation by valproic acid in zebrafish

**DOI:** 10.1007/s00427-016-0561-4

**Published:** 2016-08-26

**Authors:** Chun-xue Liu, Xiao-lan Peng, Chun-chun Hu, Chun-yang Li, Qiang Li, Xiu Xu

**Affiliations:** 1 Division of Child Health Care, Children’s Hospital of Fudan University, 399 Wanyuan Road, Shanghai, 201102 China; 2 Center for Translational Medicine, Institute of Pediatrics, Shanghai Key Laboratory of Birth Defect, Children’s Hospital of Fudan University, 399 Wanyuan Road, Shanghai, 201102 China

**Keywords:** Autism spectrum disorder, Zebrafish, Valproic acid, shank3, Transcript, Developmental expression, ANK and SAM domains

## Abstract

SHANK3 is a scaffolding protein that binds to various synaptic proteins at the postsynaptic density (PSD) of excitatory glutamatergic synapses. *SHANK3* is not only strongly implicated in autism spectrum disorders (ASD) but also plays a critical role in human Phelan-McDermid syndrome (22q13.3 deletion syndrome). Accumulated experimental evidence demonstrates that the zebrafish model system is useful for studying the functions of ASD-related gene during early development. However, many basic features of *shank3* transcript expression in zebrafish remain poorly understood. Here, we investigated temporal, spatial, and isoform-specific expression patterns of *shank3* during zebrafish development on the basis of previous researches and the differential effects of each *shank3* transcript expression after exposure to valproic acid (VPA), an ASD-associated drug. At first, we observed that both *shank3a* and *shank3b* were barely expressed at very early ages (before 24 h post-fertilization (hpf)), whereas their expression levels were increased and mainly enriched in the nervous system after 24 hpf. Secondly, all of the six *shank3* transcripts gradually increased during the first 7 hpf and then decreased. Subsequently, they exhibited a second increasing peak between 1 month post-fertilization (mpf) and adulthood. Thirdly, VPA treatment affected the isoform-specific expression of zebrafish *shank3*. In particular, the mRNA expression levels of those isoforms that contain a SAM domain were significantly increased, whereas the mRNA expression level of those which contained an ANK domain but without a SAM domain was decreased. To conclude, our findings support the molecular diversity of *shank3* in zebrafish and provide a molecular framework to understand the isoform-specific function of shank3 in zebrafish.

**Electronic supplementary material:**

The online version of this article (doi:10.1007/s00427-016-0561-4) contains supplementary material, which is available to authorized users.

## Introduction

Synaptogenesis is a key process that occurs during brain circuitry development, and inappropriate synapse formation or structure is thought to underlie a variety of neurodevelopmental disorders (van Spronsen and Hoogenraad, 2010; Guo et al., 2015). SHANK3 [SH3 and multiple ankyrin (ANK) repeat domain 3], as a member of the highly conserved SHANK/ProSAP family, is a major scaffolding protein that is enriched at the PSD of excitatory synapses (Grabrucker et al., 2011; Verpelli et al., 2011; Boccuto et al., 2013). Accumulating human genetic evidence supports a strong causal relationship between molecular defects in the *SHANK3* gene and ASD, which is a heritable, debilitating neurodevelopmental disorder (Grzadzinski et al., 2013; McGuinness and Johnson, 2013; Wang et al., 2014). Moreover, recent studies using neuronal cell cultures (Verpelli et al., 2011; Durand et al., 2012) and mouse models (Peca et al., 2011; Yang et al., 2012; Jiang and Ehlers, 2013) have demonstrated critical roles for *SHANK3* in synaptic function, social interaction and social communication, thus providing a functional link between *SHANK3* deficiency and ASD behavioral features (Boccuto et al., 2013).

Recently, much experimental evidence has demonstrated that zebrafish (*Danio rerio*) is a valuable model organism to model human brain disorders, normal and pathological social phenotypes and other ASD-like symptoms. In particular, zebrafish can be used to explore the genetic or pharmacological modulation of those diseases (Gerlai, 2010; Kalueff et al., 2013; Stewart et al., 2013; Stewart et al., 2014), making zebrafish a strong potential model organism for studying ASD gene function early in development (Pather and Gerlai, 2009; Gerlai, 2010; Gerlai, 2011; Stewart et al., 2013).

*SHANK3* appears only once in the human genome. Nevertheless, this gene is duplicated in zebrafish, appearing as *shank3a* (chromosome 18) and *shank3b* (chromosome 4) (Kozol et al., 2015). Further examination of *shank3a* and *shank3b*, as well as their different variants, will make a valuable contribution to the study of the function of human *SHANK3*.

Five intragenic promoters (Wang et al., 2011; Jiang and Ehlers, 2013; Zhu et al., 2014) and extensive alternative splice variants have been identified in mouse and human *SHANK3* (Lim et al., 1999; Wilson et al., 2003; Durand et al., 2007), which all contribute to the diversity of *Shank3* transcripts. Moreover, *Shank3* transcripts exhibit dynamic spatiotemporal domains during the developmental stage when neural segment regions are molded (Wang et al., 2014). The complexity of *Shank3* transcriptional regulation has been reported in mouse (Jiang and Ehlers, 2013; Wang et al., 2014; Zhu et al., 2014). Therefore, the clinical heterogeneity in the disorders caused by *SHANK3* mutations may be due to the differential effects of the variable locations of mutations within the coding exon of each SHANK3 isoform.

A recent study has determined the developmental expression patterns of *shank3a* and *shank3b* in zebrafish, ranging in the age from 2 to 120 hpf, and two transcripts of *shank3* gene have been verified and characterized (Kozol et al., 2015). However, six *shank3* transcripts exist according to the Ensembl database (Zv9). It is urgent to further study the developmental expression of all *shank3* transcripts in zebrafish during more developmental stages, so as to improve the expression profile characteristics. Examining and analyzing the profiles of these *shank3* transcripts may provide a foundation for the further establishment of a transgenic zebrafish model of *shank3*.

Epigenetic modifications, resulting from enzymes such as histone deacetylase (HDAC), have been shown to regulate the expression and alternative splicing of neuronal genes (Schor et al., 2009; Fischer et al., 2010). VPA is a typical example of HDAC inhibitor that was recently found to have teratogenic (interfering with early development) and neuropsychiatric side effects related to in utero exposure (Jacob et al., 2014). Moreover, fetal VPA exposure has been associated with a 3- to 46-fold increased risk of ASD (Dufour-Rainfray et al., 2010; Bromley et al., 2013; Christensen et al., 2013; Wood, 2014). Therefore, in this study, VPA was applied to investigate the alterations of the expression levels of the different *shank3* transcripts and to dissect the distinct functions of each conserved domain.

In general, examination of the transcriptional and developmental expression patterns as well as the differential effects on zebrafish *shank3* transcripts in response to VPA treatment is crucial to establish a *shank3*-knockout zebrafish model for elucidating the functions of each shank3 isoform and exploring the mechanisms underlying ASD clinical heterogeneity.

## Materials and methods

### Fish and embryo maintenance

Wild-type zebrafish (Tu) were raised and maintained under standard laboratory conditions at 28.5 °C in “system water” under a 14 h light/10 h dark cycle (Kalueff et al., 2014). Freshly fertilized eggs were collected from multiple breeding tanks containing 25 females and 25 males.

### Evolutionary comparison and verification of six zebrafish *shank3* transcripts

The zebrafish *shank3* nucleotide sequence, exon-intron structure, amino acid sequence, different transcripts and conserved domains were analyzed using the NCBI gene database (http://www.ncbi.nlm.nih.gov/gene/) and zebrafish whole genome sequence project database (http://www.ensembl.org/Danio_rerio). Protein sequences belonging to the shank3 family were identified using the NCBI BLAST program and confirmed by best reciprocal BLAST.

In particular, since the 5′ open reading frame (ORF) of *shank3a-1* was incomplete, PCR amplification was then performed on cDNA from 3-month-old zebrafish with primers designed based on conservative regions of the predicted ORF sequence (forward: 5′-GAGAGTGTATGCATGAGGGAGGCAC-3′ and reverse: 5′-GGTCTGGGTCTCCATATACAGTACCCC-3′, Supplementary Material, Fig. S1). The PCR conditions were as follows: 95 °C for 2 min, 35 cycles of 95 °C for 30 s, 56 °C for 30 s, 72 °C for 4 min 10s, and a final extension at 72 °C for 5 min. The PCR products were then cloned into a pGEM-T Easy vector (Promega) and sequenced.

To analyze *SHANK3* gene evolution and conservation, multiple alignments were performed and phylogenetic trees constructed using MEGA 6. The alignments were imported into the BoxShade Server (http://www.ch.embnet.org/software/BOX_form.html) to identify regions of high local similarity.

Based on the sequences in the Ensembl database, transcript-specific oligonucleotide primers and amplicons were generated (Supplementary Material, Fig. S1 and Table S1). cDNA from 3-month-old zebrafish was used as a template for PCR, as noted above each 2 % agarose gel in Fig. 2b.

### Spatial expression pattern of larval zebrafish *shank3a* and *shank3b* detected by whole-mount in situ hybridization

Larvae were dechorionated and placed in a mixture of fish facility system water and 0.003 % phenylthiourea at 24 hpf. Embryos were fixed and processed for WISH. Table S2 in the Supplementary Material lists the WISH primers that were used to amplify and synthesize RNA probes. The PCR products were then cloned into a pGEM-T Easy vector. Sense and antisense RNA probes were individually transcribed using linearized constructs with T7 or Sp6 polymerase (Ambion, USA) in the presence of digoxigenin (DIG, Roche, Germany)-labeled UTP using a DIG-RNA Labeling Kit (Roche, Germany). The resulting DIG-labeled antisense probes were used to label the *shank3a* and *shank3b* genes respectively, and the sense probes were used as negative controls. WISH (approximately 40 eggs/clutch) was performed as described previously (Thisse and Thisse, 2008) using 5-nitro-blue tetrazoliumchloride and bromo-4-chloro-3′-indolyl phosphate p-toluidine salt (NBT/BCIP, Vector Laboratories) as substrates (Thisse and Thisse, 2008). After WISH, the embryos were mounted in 4 % methylcellulose, and images were captured using a Leica 205C microscope.

### VPA treatments and phenotypic assessments

Embryos were dechorionated prior to drug treatment. Zebrafish embryos were exposed to a final concentration of 0.5 or 1.0 mM VPA (P4543-10G, Sigma, USA) or a control solution (0.3X Danieau’s solution), which was administered continuously from 24 hpf until 48 hpf.

Embryos exposed to VPA or the control solution were observed for morphological abnormalities at 48 hpf. The embryos were scored individually for several phenotypes, including developmental retardation, pericardial effusion, spinal curvature and reduced pigmentation. Each treatment consisted of three biological replicates with 70 embryos per replicate. During drug treatment, VPA-exposed and control zebrafish embryos were observed under a microscope for any morphologic abnormalities. The data were presented as the percentages of embryos with each defect, and the results were calculated by dividing the total number of embryos with the deformity by the total number of embryos in each replicate. Samples were collected at 48 hpf and stored at −80 °C until RNA isolation.

### Quantitative real-time PCR

Approximately 20 zebrafish embryos at 1, 3, 5, 7, 10, 13, 15, 30, and 60 days post-fertilization (dpf) were used for each condition, and each experiment was performed in triplicate. Total RNA was extracted from the embryos using TRIzol reagent (Ambion, USA). Extracted RNA was treated with DNase (Life Technologies, USA) according to the manufacturer’s protocol. Whole RNA samples were run on 2 % agarose gels to determine RNA integrity. Reverse transcription was performed with a PrimeScript™ RT Reagent Kit (TaKaRa, Japan). q-PCR was performed using a LightCycler® 480 Instrument (Roche, Germany) and SuperReal PreMix Plus (TIANGEN, China) according to the manufacturer’s recommendations. The q-PCR conditions were as follows: an initial cycle of 95 °C for 15 min; 40 cycles of 95 °C for 30 s, 60 °C for 30 s, and 72 °C for 15 s; and a final cycle of 95 °C for 1 min; followed by 40 °C for 1 min, 65 °C for 1 min, 95 °C for 1 min; and finally 40 °C for 1 min. As an internal control, a 102-bp sequence of β-actin cDNA was amplified by q-PCR using two specific primers (Supplementary Material, Table S1). The primers used in this study were designed using Primer3 and by running BLAT (https://genome.ucsc.edu/cgi-bin/hgBlat) against the zebrafish genome to confirm gene specificity and they are listed in Table S1 in the Supplementary Material (Rozen and Skaletsky, 2000). The sequences of the newly identified splice variants were annotated and deposited into the Ensembl database.

## Results

### *shank3* is a conserved gene in the vertebrate lineage that possesses multiple transcripts

The 5′ incomplete fragment of *shank3a-1* (Supplementary Material, Fig. S1) was isolated by PCR using specific primers designed to acquire the full length of the ORF. As verified by PCR and Sanger sequence analysis, *shank3a-1* was termed as the completed-length cDNA and has been studied in depth in our study.

**Fig. 1:**
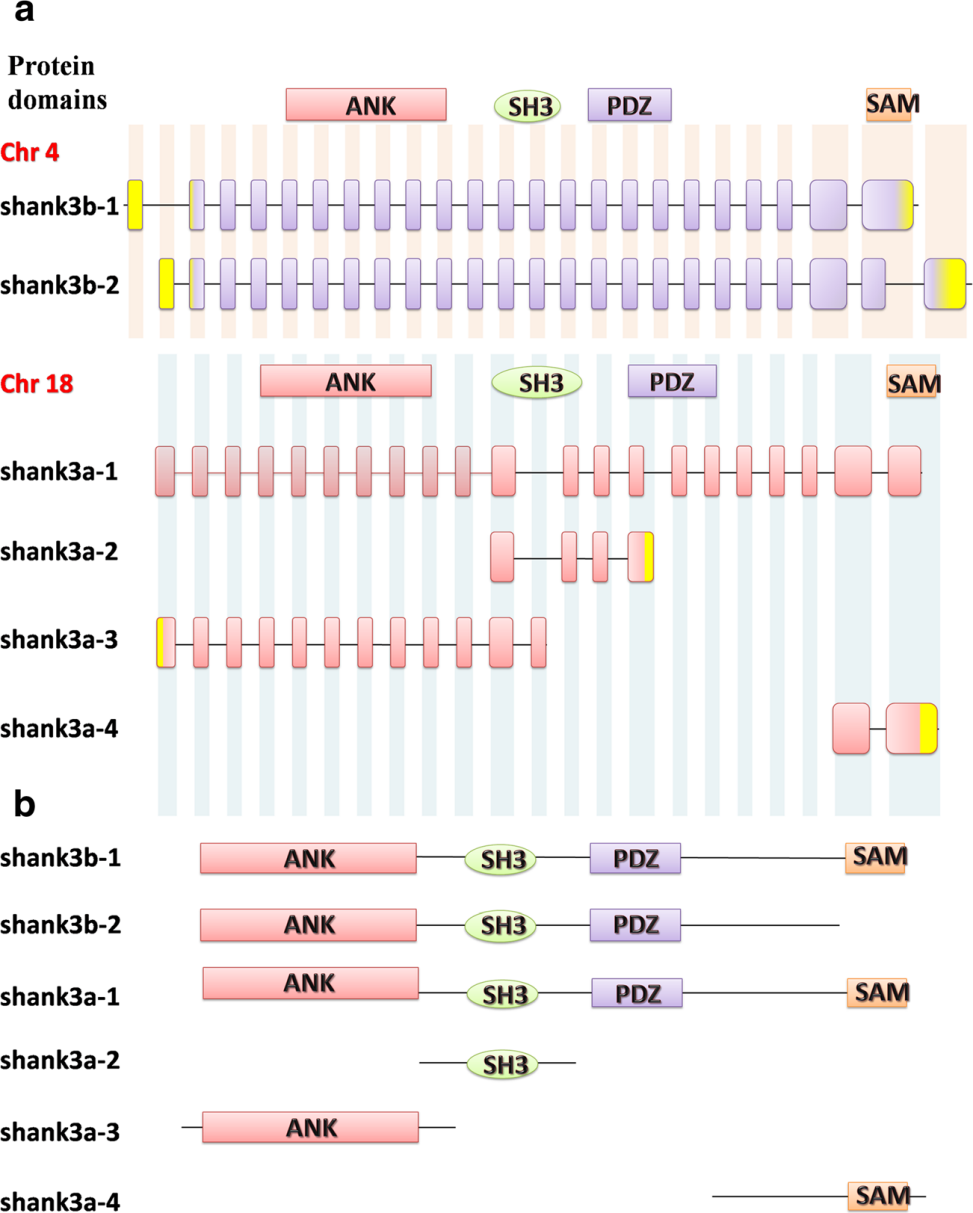
Multiple transcripts of *shank3* in zebrafish. **a** The structures of six zebrafish *shank3* transcripts are shown. *Yellow rectangles* indicate the 5′ and 3′ UTRs. Notably, the *SHANK3* gene is duplicated in zebrafish, appearing as *shank3b* (chromosome 4) and *shank3a* (chromosome 18). **b** Extensive predicted protein isoforms of zebrafish shank3. The protein domains are shown and aligned to corresponding exons (*ANK* ankyrin repeat domain, *SH3* Src homology 3 domain, *PDZ* PSD-95/discs large/ZO-1 domain, *SAM* sterile alpha motif domain

According to the NCBI BLAST Alignment results (http://blast.ncbi.nlm.nih.gov/), the longest shank3a (shank3a-1, our study) and shank3b (shank3b-1) protein sequences of *Danio rerio* (Fig. 1a) exhibit a high level of amino acid identity (55 and 59 %, respectively) with the corresponding *Homo sapiens* SHANK3 protein (NP_277052.1). In particular, the four typical conserved domains (ANK, SH3, PDZ, and SAM domain) of shank3 exhibit at least 80 % amino acid identity (Supplementary Material, Fig. S2). These findings indicate that human SHANK3 is highly conserved in zebrafish and that zebrafish might be a strong potential model for studying human SHANK3.

**Fig. 2:**
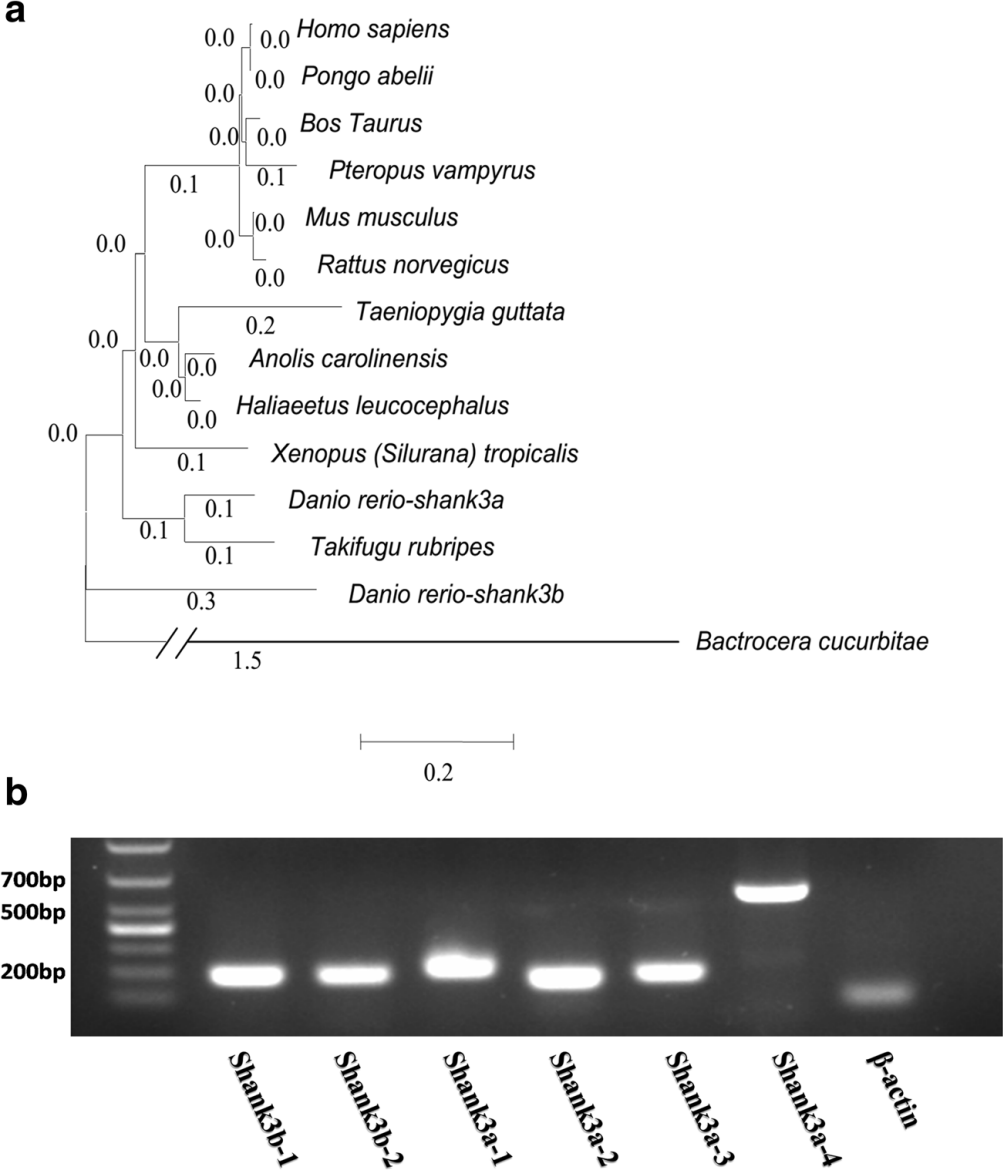
Phylogenetic tree of evolutionary relationship of SHANK3 proteins. **a** Diverse animal phyla were identified by a best reciprocal BLAST search with human SHANK3 and are mapped onto a phylogenetic tree. All of the sequences are available from the NCBI protein database with the exception of zebrafish shank3a, which was obtained from the Ensemble protein database and our study. *Homo sapiens* (NP_277052.1), *Pongo abelii* (XP_009232827.1), *Rattus norvegicus* (NP_067708.1), *Mus musculus* (NP_067398.2), *Bos Taurus* (XP_010804153.1), *Taeniopygia guttata* (XP_012432256.1), *Xenopus (Silurana) tropicalis* (XP_002941881.2), *Danio rerio shank3b* (XP_001919745.1), *Bactrocera cucurbitae* (XP_011184638.1), *Pteropus vampyrus* (XP_011382480.1), *Takifugu rubripes* (XP_003967184.1), *Anolis carolinensis* (XP_008121382.1), *Haliaeetus leucocephalus* (XP_010564365.1), and *Danio rerio shank3a* (ENSDART00000139505 and our study). The tree was constructed using the neighbor-joining methods as implemented in the MEGA 6 package. Clade robustness was measured by the bootstrap method with 1000 replicates. The lengths of the lines are proportional to the evolutionary distances from branching points. **b** Transcript-specific oligonucleotide primers and amplicons were generated to confirm the existence of the key fragment of six predicted transcripts of *shank3* in the Ensembl database. cDNA from 3-month-old zebrafish was used as a template for PCR, as noted above each 2 % agarose gel. The housekeeping gene β-actin was used as an internal reference

To further examine the orthologs between zebrafish *shank3* and other species, we performed phylogenetic analysis to compare shank3 of *Danio rerio* with that of *Homo sapiens* and 11 other species by constructing a phylogenetic tree (Fig. 2a). The obtained numbers represented evolutionary relationships, with larger numbers indicating greater genetic differences. In vertebrate animals, the genetic distance was not greater than “0.3”; however, in invertebrates (such as *Bactrocera cucurbitae*), the distance was as high as “1.5” (Fig. 2a). Therefore, this phylogenetic analysis revealed a highly conserved pattern in mammals, rodents, and other vertebrates, implying that shank3 is unique to the vertebrate lineage.

However, the alternative splicing of *shank3* in zebrafish has not been fully characterized (Gauthier et al., 2010). Based on the sequences in the Ensembl database, each transcript of zebrafish *shank3* has some unique sequences that are either in the exon or intron of other transcripts. We were able to generate transcript-specific oligonucleotide primers and amplicons to confirm the existence of *shank3* splice variants (Supplementary Material, Fig. S1). The amplification results confirmed that the key sequences of the six zebrafish *shank3* transcripts did exist (Fig. 2b).

### Spatial and temporal expressions of *shank3* in zebrafish

**Fig. 3:**
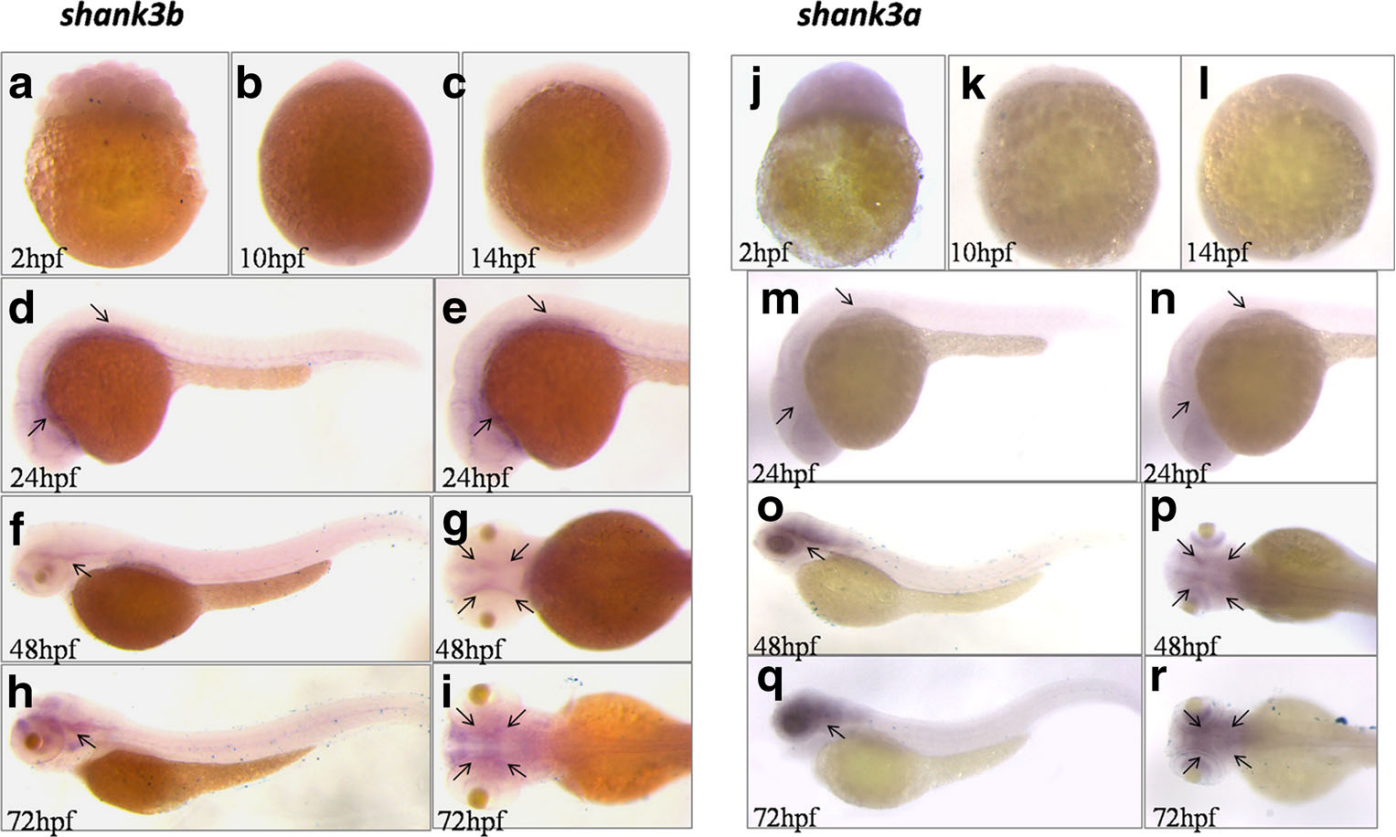
Analysis of region-specific expression of *shank3a* and *shank3b* in wild-type zebrafish by WISH. **a**–**i** WISH analysis of the level and pattern of *shank3a* and *shank3b* expressions at 2, 10, 14, 24, 48, and 72 hpf were showed. The *black arrow heads* in **d**–**i** and **m**–**r** show the expression signals in the zebrafish larva brain. Obviously, the expression of both of these isoforms was mainly concentrated in the nervous system of zebrafish larvae, particularly in the anterior-ventral forebrain

To determine the spatial and temporal expression patterns of *shank3a* and *shank3b* in zebrafish, we chose the longest zebrafish *shank3a* transcript (*shank3a-1*) and *shank3b* transcript (*shank3b-1*) to perform the whole mount in situ hybridization at 2, 10, 14, 24, 48, and 72 hpf. We found that both of them exhibited a specific pattern of concentrated expression in the zebrafish larval brain. At early ages (before 24 hpf, Fig. 3a–c, j–l), both *shank3a* and *shank3b* were barely expressed, whereas their expression levels increased after 24 hpf (Fig. 3d–i, m–r) and tended to increase throughout development in zebrafish larvae. In addition, the expression levels of *shank3a* and *shank3b* on 24 hpf were similar, whereas on 48 and 72 hpf, *shank3a* displayed a higher expression level than *shank3b*. Moreover, the expressions of both of these isoforms were mainly concentrated in the nervous system of zebrafish larvae, particularly in the anterior-ventral forebrain.

**Fig. 4:**
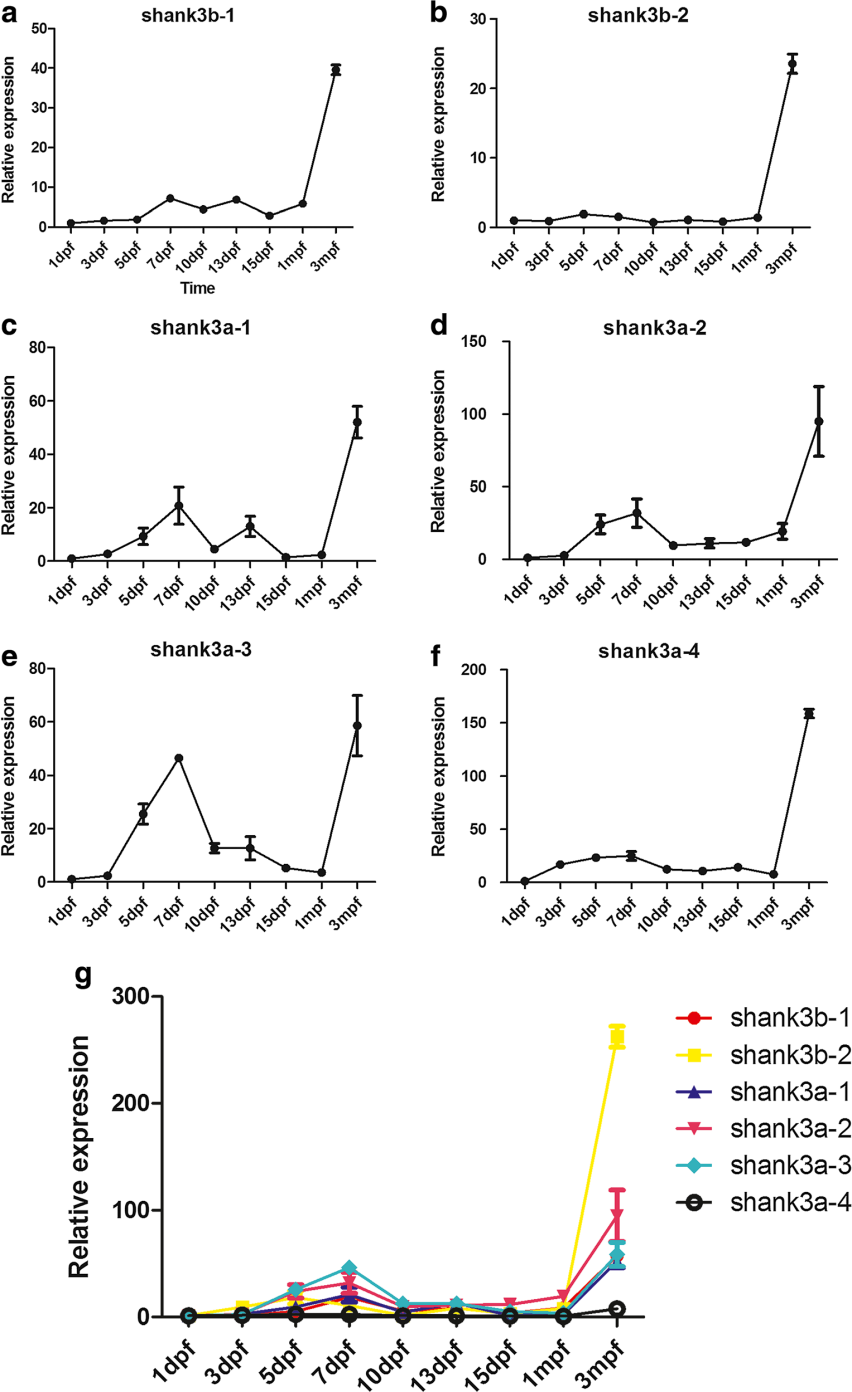
Differential expression of zebrafish *shank3* transcripts throughout development. **a**–**g** The expression of six *shank3* transcripts of zebrafish from 1 day to 3 months post-fertilization was determined by q-PCR. The crossing point (CP) values of each transcript were normalized to that of a housekeeping gene, β-actin. The *error bars* denote the standard deviations from three RNA sample replicates for each developmental time point. All data are shown as the means ± SEM

To further distinguish the different developmental profiles of each shank3 isoform, isoform-specific q-PCR was performed using whole brains from wild-type zebrafish on nine different developmental stages (Fig. 4g). Remarkably, the expression of all zebrafish *shank3* transcripts gradually increased over the first 7 dpf (Fig. 4g, a–f). The most obvious increasing trend in *shank3* expression was observed from 3 to 7 dpf, correlating with the WISH expression pattern and the time frame for synaptogenesis. A peak in *shank3* expression was observed on approximately 7 dpf. Notably, all of the *shank3* transcripts displayed a gradual decrease in expression after 7 dpf, and their expression levels, with the exception of that of *shank3b-2* (Fig. 4b), decreased by at least 50 % compared with the peak point by 15 dpf. In addition, these transcripts displayed a second increasing peak between 1 mpf and adulthood. The expression levels of *shank3b-1* (Fig. 4a), *shank3a-1* (Fig. 4c) and *shank3a-3* (Fig. 4e) markedly increased from 5 to 7 dpf. In contrast, the *shank3b-2* (Fig. 4b) and *shank3a-4* (Fig. 4f) expression levels were relatively stable before adulthood.

### Isoform-specific expression pattern of *shank3* during development

**Fig. 5:**
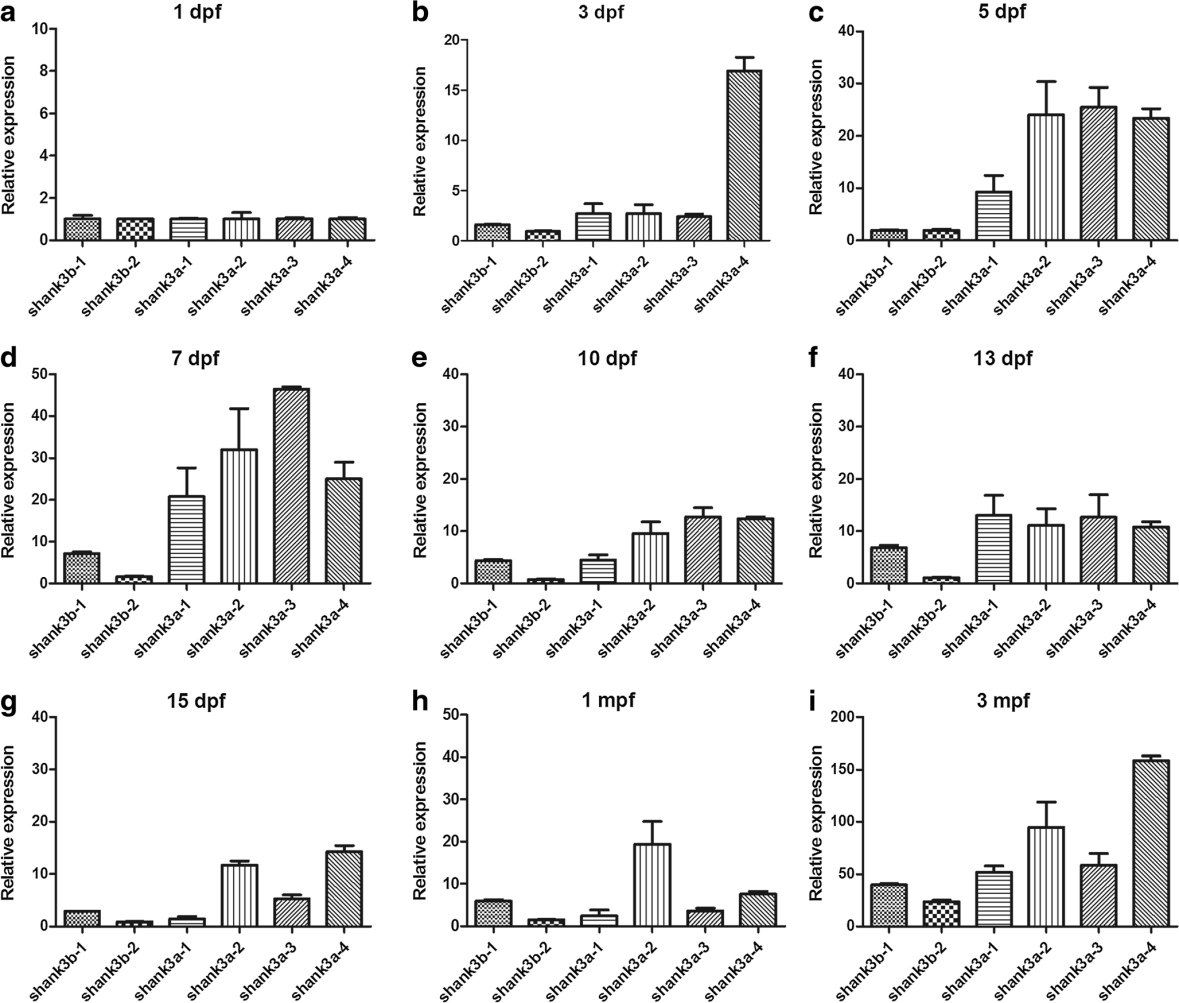
Isoform-specific pattern during the same period. **a**–**i** Comparisons of the expression patterns of six different shank3 isoforms in the zebrafish brain at the same stage are shown. The crossing point (CP) values of each isoform were normalized to that of a housekeeping gene, β-actin. The *error bars* denote the standard deviations from three RNA sample replicates for each developmental time point. All data are shown as the means ± SEM

To perform an in-depth analysis of the differential expression of each isoform at the same age stage, the isoform-specific expression of zebrafish *shank3* was then examined (Fig. 5). The expression levels of *shank3b* transcripts (*shank3b-1* and *shank3b-2*) were low throughout development; whereas all *shank3a* transcripts (*shank3a-1, shank3a-2, shank3a-3* and *shank3a-4*) displayed relatively higher expression levels. On 1 dpf, all of the transcripts showed relatively lower expression levels (Fig. 5a). However, during the period from 3 to 10 dpf (Fig. 5b–e), the expression levels of all *shank3a* transcripts were obviously higher than that of *shank3b*, which is consistent with the WISH results (see above). Moreover, the difference between *shank3b-1* and *shank3b-2* was not significant from 1 to 5 dpf (Fig. 5a–c), whereas after 7 dpf, the expression of *shank3b-1* was higher than that of *shank3b-2*.

### The ANK and SAM domains of zebrafish shank3 may display the opposite effect following treatment with VPA

**Fig. 6:**
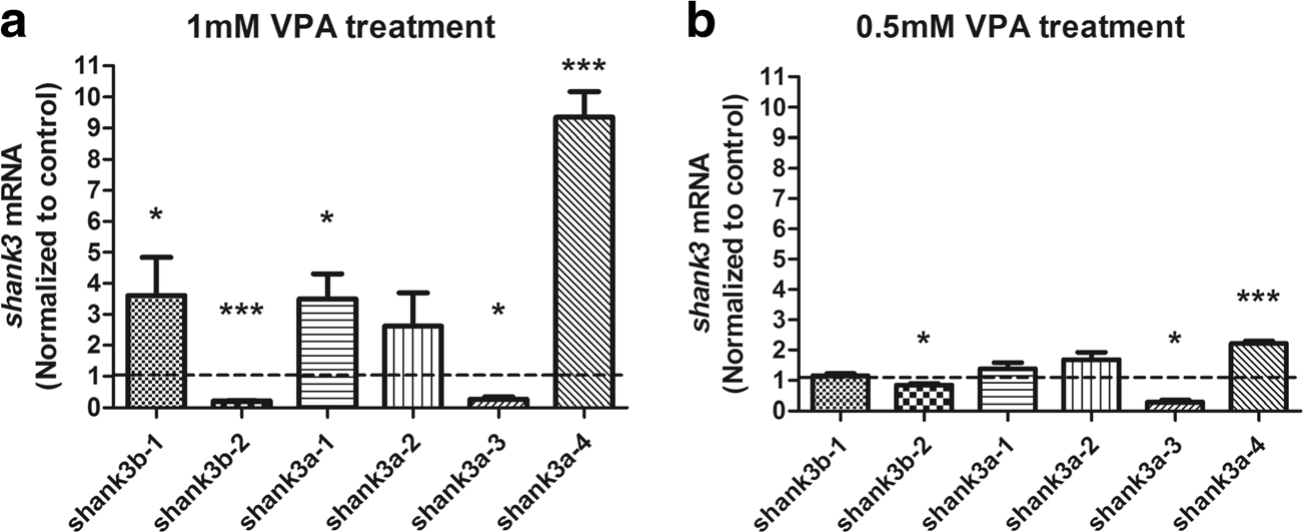
Isoform-specific expression of zebrafish shank3 induced by VPA compared with the control. **a**, **b** q-PCR analysis of zebrafish *shank3* transcripts at 48 hpf after treatment with 1 or 0.5 mM VPA for 24 h. All data are shown as the means ± SEM. The statistical significance values, such as *P < 0.1, **P < 0.01, and ***P < 0.005, of differences compared with the control group were determined through two-tailed *t* test (*n* = 15 in each group)

To examine whether different *shank3* transcripts were subject to HDAC, zebrafish embryos were treated with VPA. The concentrations that caused teratogenic effects, as detailed in the literature, varied among studies but generally ranged from 0.1 to 3.0 mM (Li et al., 2009; Selderslaghs et al., 2009; Brannen et al., 2010; Terbach et al., 2011; Cowden et al., 2012; Teixido et al., 2013). Thus, an initial dose-response experiment was conducted to determine the effects of two different VPA concentrations, 1 and 0.5 mM, on zebrafish embryos (Fig. 6). The VPA-treated zebrafish embryos displayed numerous morphological abnormalities, and those exposed to a higher concentration displayed more deformities. Most embryos exposed to 1 mM VPA exhibited phenotypic abnormalities, including developmental retardation (100 %, 70/70), reduced pigmentation (100 %, 70/70), spinal curvature (43 %, 30/70) and pericardial effusion (64 %, 45/70, Supplementary Material, Figs. S3A and B). Additionally, embryos that exposed to 0.5 mM VPA displayed relatively fewer defects, such as developmental retardation (100 %, 70/70), reduced pigmentation (83 %, 58/70), spinal curvature (20 %, 14/70) and pericardial effusion (40 %, 28/70, Supplementary Material, Figs. S3C and D).

Notably, the VPA treatment exhibited varying effects on different *shank3* transcripts. In the zebrafish embryos treated with 1 mM VPA, the expressions of all transcripts containing the SAM domain (*shank3b-1, shank3a-1*and *shank3a-4*, Fig. 1b) were significantly increased (Fig. 6a), whereas of the expressions of *shank3b-2* and *shank3a-3* (Fig. 1b), which contained an ANK domain but without the SAM domain, were remarkably decreased (Fig. 6a). The opposite direction was also replicated in lower VPA concentrations (0.5 mM, Fig. 6b). In summary, our data suggest that the ANK and SAM domains of shank3 are more susceptible to VPA and are crucial for the normal function of shank3.

## Discussion

Our study demonstrated that zebrafish shank3 was unique to the vertebrate lineage (Fig. 2a) and possessed an ortholog of human SHANK3 that has retained the conserved ANK, SH3, PDZ and SAM domains (Supplementary Material, Fig. S2). Comprehensive and systematic experiments were performed to analyze the isoform-specific expression of shank3, which displayed temporal and spatial specific patterns. In particular, VPA could regulate the expression of *shank3* in zebrafish, and *shank3* transcripts containing a SAM domain displayed significantly decreased expression patterns, whereas those containing an ANK domain but without the SAM domain showed the opposite trend, supporting the hypothesis that each isoform has distinct functions because of the different features of each domain.

Taken together, these findings will enhance the current knowledge of the molecular diversity in the brain and provide insights into the phenotypic heterogeneity caused by various *SHANK3* defects in humans.

### The expression profiles of *shank3* may be linked with synaptic development

*Shank3* expression has been widely characterized in mammalian animal models (Jiang and Ehlers, 2013; Wang et al., 2014), and the spatial expression patterns of *shank3a* and *shank3b* at 48 hpf in zebrafish have also been determined (Kozol et al., 2015). On the basis of previous research, we further validated the expression patterns of more developmental stages. The results revealed that zebrafish *shank3* was mainly expressed in the nervous system, particularly in the anterior-ventral forebrain (Fig. 3d–i, m–r). Over the first 3 days, almost all *shank3* transcripts displayed a low level of expression (Fig. 4g); thereafter, a rapid increase in expression that corresponded with the time frame for synaptogenesis was observed. As a scaffolding protein, shank3 played a key role in synaptic formation, maturation, maintenance, and transmission. The increasing expression over the first several days corresponded with the rapid development of the nervous system, which indirectly reflected the increasing head circumferences in children with age. The increase in brain size over time was due to the early growth of gray matter followed by the pruning of dendrites and synaptic structures, combined with major growth of white matter as it underwent myelination (Geschwind and Levitt, 2007). Shaw et al. (Shaw et al., 2006) suggested that cognitive skills were more significantly correlated with the developmental trajectory of brain volumes over time than with static brain volume.

Our results also demonstrated that the expressions of *shank3* transcripts declined afterward, which largely coincided with the data obtained from mouse studies (Wang et al., 2014). Wang et al. examined the developmental expression of five *Shank3* transcripts in mouse brains with different ages and observed a peak of expression that occurred between 2 and 4 weeks. However, in our results, the first peak was observed on approximately 7 dpf and the second peak was detected during adulthood. In addition, in mouse studies (Wang et al., 2014), two transcripts (*Shank3a* and *Shank3b*) displayed similar trends to those of *shank3b-1* and *shank3b-2*, respectively, in our study. Interestingly, in-depth investigations into the protein structures revealed that these mouse and zebrafish transcripts share the same conserved domains. The other four *shank3* transcripts (*shank3a-1*, *shank3a-2*, *shank3a-3* and *shank3a-4*) in zebrafish differed from those in the mouse, which may reflect functional differences. Therefore, a more thorough analysis of these different transcripts would compensate for insufficiencies in mouse models.

Two recent studies have analyzed the *shank3* gene in zebrafish (Gauthier et al., 2010; Kozol et al., 2015). Both studies used morpholinos to knock down the expression of *shank3a* and *shank3b* (Kozol et al., 2015) or *zs3.1* and *zs3.2* (Gauthier et al., 2010), systematically clarifying the functions of the two *shank3* transcripts. Upon performing a BLAST search of the morpholino oligonucleotides used in their studies (Supplementary Material, Fig. S4), we found that *shank3a* (Kozol et al., 2015) corresponded to *shank3a-1* and *shank3a-3* in our study and that *shank3b* (Kozol et al., 2015) corresponded to *shank3b-1 and shank3b-2*. Thus, only some of the *shank3* transcripts were likely knocked down, whereas the others remained unaffected. Furthermore, in the study of Gauthier et al., only four transcripts (*shank3b-1*, *shank3b-2*, *shank3a-1* and *shank3a-2*) were knocked down (Gauthier et al., 2010). A further study of the functions of the remaining transcripts is warranted. In addition, the studies of these transcripts are currently underway in our laboratory.

Parenthetically, each shank3 isoform has a unique combination of protein domains. Full-length shank3a-1and shank3b-1 contain all four domains (ANK, SH3, PDZ, and SAM), which allow for their interactions with all possible interacting proteins. Other isoforms with different combinations of functional domains interact with different subsets of synaptic proteins. For instance, the C-terminal truncated shank3a-3 (Fig. 1b) can only interact with α-fodrin but not with mGluRs, Homer1 or other partners. Because each protein domain mediates a unique complement of protein-protein interactions (Hayashi et al., 2009), each shank3 isoform likely has distinct functions.

Taken together, these results provide evidence to support the findings that different shank3 isoforms have distinct functions at synapses and are associated with different clinical presentations. However, the exact mechanism regulating shank3 transcription in zebrafish remains to be investigated in the future.

### The SAM and ANK domains may be a key connection between shank3 and VPA

The epigenetic modification of DNA by the methylation of cytosine in CpG dinucleotides and histone modification represented a common mechanism to regulate the gene expression (Collas, 1998). VPA is a known HDAC inhibitor that could activate genes through reprogramming of genome expression and by causing hyperacetylation of induced genes (Milutinovic et al., 2006).

In this study, we demonstrated that VPA treatment altered *shank3* mRNA expression during zebrafish development. To the best of our knowledge, this is the first study evaluating the effects of VPA on *shank3* transcript expression in zebrafish. Treatment with VPA during embryonic development induced a delayed development in hatching and an increased number of malformations. The toxic responses to VPA (1 and 0.5 mM) were dose-dependent (Terbach et al., 2011).

Notably, the expression levels of *shank3b-1*, *shank3a-1* and *shank3a-4* significantly increased after treatment with 1 mM VPA (Fig. 6a). Further analyses of their protein structures revealed that the three transcripts all contained a SAM domain (Fig. 1b). The SAM domain was next to the C terminus of shank3 and these proteins bound to each other in a homomeric and heteromeric manner, indicating that shank3 proteins could multimerize in a tail-to-tail manner (Sheng and Kim, 2000).

Another interesting finding was that the expression levels of *shank3b-2* and *shank3a-3*, which lacked the SAM domain but contained the ANK domain, were markedly decreased after exposure to both 1 and 0.5 mM VPA (Fig. 6a). However, full length shank3a-1 and shank3b-1 isoforms that encoded all protein interaction domains exhibited the reverse effect. One possible explanation was that the SAM domain likely offsetted the increasing effect. The ANK domain associated with the actin-based cytoskeleton and was conducive to dendritic spine development (Lim et al., 1999; Jiang and Ehlers, 2013). α-Fodrin has been shown to interact with the N-terminal ANK repeats of shank3 (Bockers et al., 2001). In addition, ANK repeats exhibited secondary structures in the form of pairs of anti-parallel α-helices that were linked by a variety of β-hairpin motifs. However, these repeats did not prefer specific motifs, nor did they recognize consensus sequences of the target molecules (Sedgwick and Smerdon, 1999; Bockers et al., 2001).

Shank3 contains multiple domains for protein-protein interactions, but understanding of their functional significance for each domain is lacking (Sheng and Kim, 2000). Our results suggested that VPA selectively altered the expression of *shank3* transcripts containing the SAM and ANK domains and implied a functional correlation for the Shank3 proteins with these domains.

The complexity of the shank3 transcript structure indicates that the point mutations, translocations, and intragenic deletions of *shank3* observed in ASD patients are isoform-specific (Jiang and Ehlers, 2013). Further studies are necessary to determine the functions of each isoform in vivo and their relevance to synaptic and behavioral phenotypes. Understanding the unique function of each *shank3* transcript will undoubtedly be beneficial for future drug screening.

## Electronic supplementary material

ESM 1 (DOC 8107 kb)
