# Supplementary material for: Developmental profiling of ASD-related shank3 transcripts and their differential regulation by valproic acid in zebrafish
Source: Dev Genes Evol. 2016 Aug 26;226(6):389–400. doi: 10.1007/s00427-016-0561-4 (PMC5099374; doi:10.1007/s00427-016-0561-4)
Supplement: Supplementary file 1 — (DOC 8107 kb) [file 427_2016_561_MOESM1_ESM.doc]

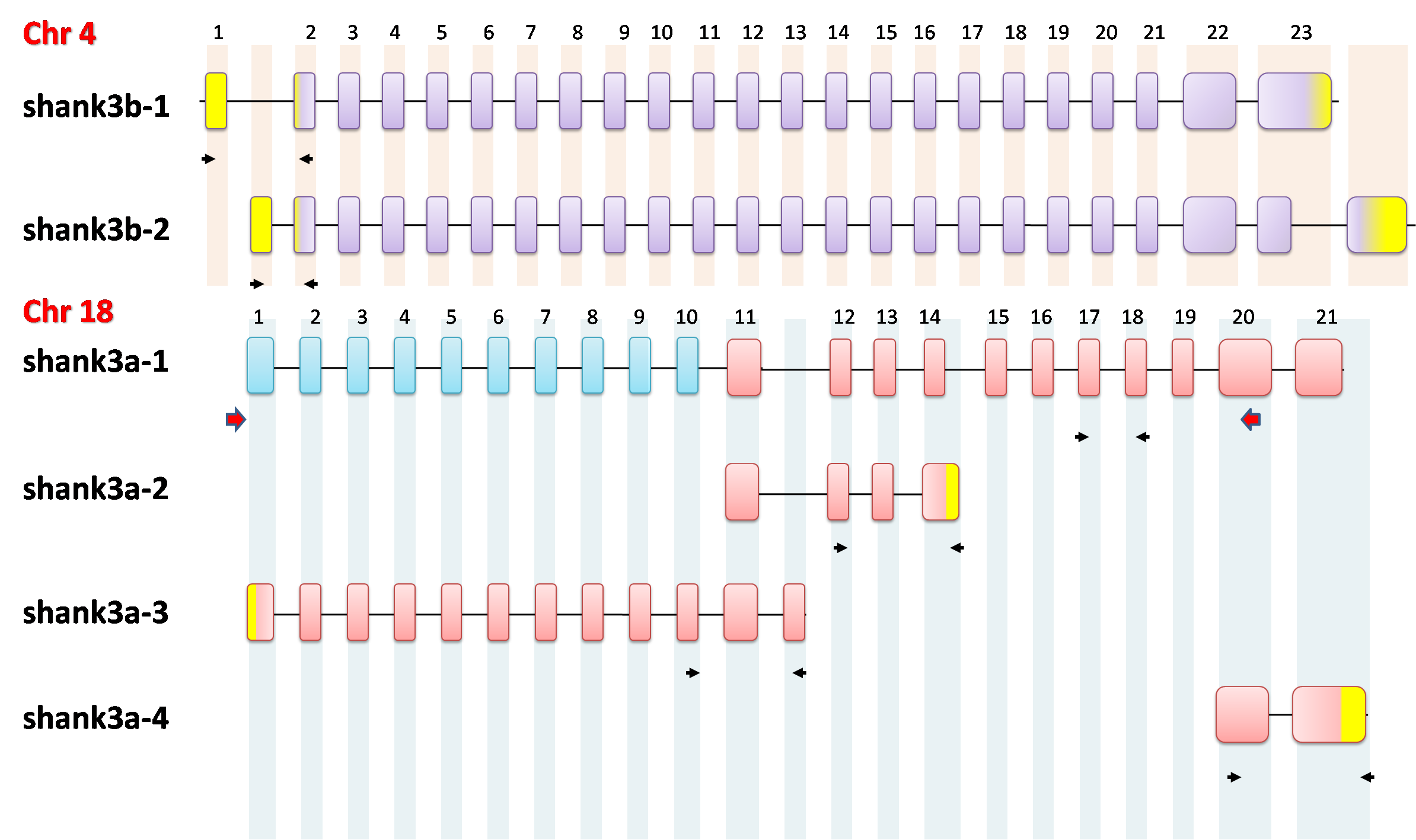


**Supplementary Material, Fig. S1** Each isoform of zebrafish shank3 has some unique sequences that fall in the exon or intron of other isoforms. The left and right isoform specific primers were design in these regions. In lower panel, positions of isoform-specific primers used in this study are indicated. In addition, specific primers (red arrow) were designed to amplify the 5’ incompleted sequences of shank3a-1.

Homo 1 M-----------------------------------------------------------
Mus 1 M-----------------------------------------------------------
Bos 1 P-----------------------------------------------------------
Takifugu 1 M-----------------------------------------------------------
Da-shank3b 1 MPISPPADGKHEALDRPWQQHAP-------------------------------------
Da-shank3a 1 MPVSAGSGKHEPSQPRDRPQQQPEVPSEPPSALPSVSPSLSLPLSLPLLIYSRPAQPLPR
consensus 1 .


Homo 2 ---------------------------DGPGASAVVVRVG------IPDLQQTKCLRLDP
Mus 2 ---------------------------DGPGASAVVVRVG------IPDLQQTKCLRLDP
Bos 2 ----------SELGAEAGVRGLRSRAGTRAAPRASVPRAGREARSAHDRAPLQKCLRLDP
Takifugu 2 ---------------------------EEPTGNTVVIRIG------IPDLQQTKCLKFNV
Da-shank3b 24 --TNGNHGDDSIRTSPGSKCGS--EPMEDLHGNAVVIRIG------IPDLQQTKCLRLDL
Da-shank3a 61 HLSSSTSRGESARSPHADVCRAPAGPMEEPQGNTLVVRIG------IPDLQQTKCMKFNV
consensus 61 . . ........*.*.* .......**.....


Homo 29 AAPVWAAKQRVLCALNHSLQDALNYGLFQPPSRGRAGKFLDEERLLQEYPPNLDTPLPYL
Mus 29 TAPVWAAKQRVLCALNHSLQDALNYGLFQPPSRGRAGKFLDEERLLQDYPPNLDTPLPYL
Bos 52 AAPVWAAKQRVLCALNHSLQDALNYGLFQPPSRGRAGKFLDEERLLQEYPPNLDTPLPYL
Takifugu 29 DAPIWLSKRRILCTLNQSLKDVLNYGLFQPAYNGKAGKFLDEERQLREYPFPSLAPVPYL
Da-shank3b 74 EAPVWVCKQRVLVTLTQSLTDVLNYGLYLPAFNGRAGKFLDEERLLREYPLPTVTPVPYL
Da-shank3a 115 ESPIWLSKQRILCTLNQSLKDVLNYGLFLPAYNGKAGKFLDEQRTLKEYPLPAVSPVPYL
consensus 121 ..*.*..*.*.*. *. **.* *****..* . *.*******.*.*..**. ...*.***


Homo 89 EFRYKRRVYAQNLIDDKQFAKLHTKANLKKFMDYVQLHSTDKVARLLDKGLDPNFHDPDS
Mus 89 EFRYKRRVYAQNLIDDKQFAKLHTKANLKKFMDYVQLHSTDKVARLLDKGLDPNFHDPDS
Bos 112 EFRYKRRVYAQNLIDDKQFAKLHTKANLKKFMEYVQLHSTEKVARLLDKGLDPNFHDPDS
Takifugu 89 EFRYKRRVYTQTHLDEKQLSKLHTKANLKKFMEHVQQRNIEKVSRFLDKGLDPNFHDPDT
Da-shank3b 134 EFRYKRRVYTQSHVDDKQLAKLHTKANLKKFMEYVQQRCVDKVCRFLEKGLDPNFHDSDS
Da-shank3a 175 EFRYKRRVYTQAHLDEKQLSKLHTKANLKKFMEYVQQRNVEKVSKFLEKGLDPNYHDPDT
consensus 181 ********* *. .*.** .************..** ....**.. *.******.**.*.


Homo 149 GECPLSLAAQLDNATDLLKVLKNGGAHLDFRTRDGLTAVHCATRQRNAAALTTLLDLGAS
Mus 149 GECPLSLAAQLDNATDLLKVLRNGGAHLDFRTRDGLTAVHCATRQRNAGALTTLLDLGAS
Bos 172 GECPLSLAAQLDDATDLLKVLKNGGAHLDFRTRDGLTAVHCATRQRNAAALTTLLDLGAS
Takifugu 149 GECPLTLVAKLEGCADLIKVLKNGGAHLDFRTKDGITALHKAVRSKNHTALITLLDLGAS
Da-shank3b 194 GESPLTLVAQLDTCADLIKVLRSGGAHLDFRTRDGLTALHKAAQTHNHVALTTLLDLGAS
Da-shank3a 235 GECPLTMASQLEGCAELIKVLKNGGAHLDFRTRDGITALHKAVRTKNHTALITLLDLGAS
consensus 241 **.**.....*. .*.***..*********.**.**.* *....* .**.********


Homo 209 PDYKDSRGLTPLYHSALGGGDALCCELLLHDHAQLGITDENGWQEIHQACRFGHVQHLEH
Mus 209 PDYKDSRGLTPLYHSALGGGDALCCELLLHDHAQLGTTDENGWQEIHQACRFGHVQHLEH
Bos 232 PDYKDSRGLTPLYHSALGGGDALCCELLLHDHAQLGTTDENGWQEVHQACRFGHVQHLEH
Takifugu 209 PDYKDSRGLTPLYHSSMVGGDPYCCELLLHDHAQVGCVDENGWQEIHQACRYGHVQHLEH
Da-shank3b 254 PDYKDSRGLTPLYHSAMVGGDPYCCELLLYDHAQLGYSDENGWQEIHQACRHGNVQHLEH
Da-shank3a 295 PDYKDSRGLTPLYHSSMVGGDPYCCELLLHDHAQVGCMDENGWQEIHQACRHGHVQHLEH
consensus 301 ***************.. *** ******.****.* .*******.*****.*.******


Homo 269 LLFYGADMGAQNASGNTALHICALYNQESCARVLLFRGANRDVRNYNSQTAFQVAIIAGN
Mus 269 LLFYGANMGAQNASGNTALHICALYNQESCARVLLFRGANKDVRNYNSQTAFQVAIIAGN
Bos 292 LLFYGADMGAQNASGNTALHICALYNQESCARVLLFRGANKDVRNYNSQTAFQVAIIAGN
Takifugu 269 LLFYGADMSAQNASGNTALHVCALYNQESCARVLLFRGANKEIKNYNSQTAFQVAIIAGN
Da-shank3b 314 LLFYGAEMSAQNASGNTALHLCALYNQEGCARVLLFRGANKEIKNYNNQTAFQVAIIAGN
Da-shank3a 355 LLFYGADMSAQNASGNTALHVCALYNQDSCARVLLFRGANKEIKNYNSQTAFQVAIIAGN
consensus 361 ******.* ***********.******..***********....***.************


Homo 329 FELAEVIKTHKDSDVVPFRETPSYAKRRRLAGPSGLASPRPLQRSASDINLKG--EAQPA
Mus 329 FELAEVIKTHKDSDVVPFRETPSYAKRRRLAGPSGLASPRPLQRSASDINLKG--D-QPA
Bos 352 FELAEVIKTHKDSDVVPFRETPSYAKRRRLAGPSALASPRPLQRSASDINLKG--EVQPA
Takifugu 329 FDLAEIVKVHKSSDVVPFRETPSYTNRRRATG--PLPSPRSLLRSASDNNLNGDHDRIHS
Da-shank3b 374 FDLAEIIKIHKTSDVVPFRETPSYSSRRRAVC---VSPRRSLMRSASDNALD---ETLTA
Da-shank3a 415 FDLAEIIKTHKASDVVPFRETPSYTNRRRVTGGDTLTSSRSLPRSASDNNLNNVADASHA
consensus 421 *.***..*.**.************..***..........* *.***** .*.. . ...

Homo 387 ASPGPSLRSLPHQLLLQRLQ--EEKDRDRDADQESNISGPLAGRAGQSKISP-----SGP
Mus 386 ASPGPTLRSLPHQLLLQRLQ--EEKDRDRDGELENDISGPSAGRGGHNKISP-----SGP
Bos 410 ASPGPSLRSLPHQLLLQRLQ--EEKDRDRDGDQQND-SGPATGRGSQSKISPKPLRTSSP
Takifugu 387 HSPVPSLRSLPAFGRHGEIP--DSSLQSTGSSRSSHSRSPSLHHMHEEDKPVPRRSHSHG
Da-shank3b 428 PSPAPSLRSLPPLEP-------DDTVPSQRSPQAAHTHTRSLRRHTR-------------
Da-shank3a 475 HSPVPSLRSLPPLAHSGIDPAADGSLQSTGSSLSSHSRSPSLQRVTEEASVLAGRRHMP-
consensus 481 .**.*.*****......... ..... ..... ............ .... . .


Homo 440 G------GPGPAPGP-GPAPPAPPA--------------------PPPRGPKRKLYSAVPGRKFIAVKAHSPQ
Mus 439 G------GSGPAPGP-GPASPAPPA--------------------PPPRGPKRKLYSAVPGRKFIAVKAHSPQ
Bos 467 GRPRLPVSPRSSVGP-TPPQPSPPR----------------TVVLLGPRKLYSAVPGRKFIAVKAHSPQ
Takifugu 445 YPHGHGHRGRLSPGS-MQRDPSPPHHTP-PALAGSRGPKRKLYSAVPGRTFIVVKPYTPQ
Da-shank3b 468 ------SGGHLSPGSPVQREPSPP----------------------AVPRGPKRRLYSAVPGRTFIAINSHTPQ
Da-shank3a 534 LMHSH-SRGRLSPGT-VQRDPSPPPVGPSHTLSGARGPKRKLYSAVPGRTFIVVKPYSPQ
consensus 541 . ......*. . ..*.** .....*.******** **......**


Homo 486 GEGEIPLHRGEAVKVLSIGEGGFWEGTVKGRTGWFPADCVEEVQMRQHDTRPETREDRTK
Mus 485 GEGEIPLHRGEAVKVLSIGEGGFWEGTVKGRTGWFPADCVEEVQMRQYDTRHETREDRTK
Bos 519 GEGEIPLHRGEAVKVLSIGEGGFWEGTVKGRTGWFPADCVEEVQMRQYDARHETREDRTK
Takifugu 503 GEGEIQLNRGERVKVLSIGEGGFWEGTVKGRTGWFPADYVEEVQMRQYDPRLETREDRTK
Da-shank3b 514 GEGEITLNRGERVKVLSIGEGGFWEGSVKGRTGWFPAHCVEEVQMRQYDPRLETREDRTK
Da-shank3a 592 GEGEIQLNRGERVKELKVS-----------------------------------------
consensus 601 *****.* *** **.*................................. . ........


Homo 546 RLFRHYTVGSYDSLTSHSDYVIDDKVAVLQKRDHEGFGFVLRGAKAETPIEEFTPTPAFP
Mus 545 RLFRHYTVGSYDSLTSHSDYVIDDKVAILQKRDHEGFGFVLRGAKAETPIEEFTPTPAFP
Bos 579 RLFRHYTVGSYDSLSAHSDYVIDDKVAVLQKRDHEGFGFVLRGAKAETPIEEFTPTPAFP
Takifugu 563 RLFRHYTVGSYDNFTSYSDYIIEEK NAVLQKKESEGFGFVLRGAKAETPIEEFTPTPAFP
Da-shank3b 574 RLFRHYTVGSYDNYTSYSDYVIEEKSATLQKRDSEGFGFVLRGAKAETPIEEFTPTPAFP
Da-shank3a 611 ------------------------------------------------------------
consensus 661 ............................................................


Homo 606 ALQYLESVDVEGVAWRAGLRTGDFLIEVNGVNVVKVGHKQVVALIRQGGNRLVMKVVSVT
Mus 605 ALQYLESVDVEGVAWRAGLRTGDFLIEVNGVNVVKVGHKQVVGLIRQGGNRLVMKVVSVT
Bos 639 ALQYLESVDVEGVAWRAGLRTGDFLIEVNGVNVVKVGHKQVVALIRQGGNRLVMKVVSVT
Takifugu 623 ALQYLESVDVEGVAWRAGLRTGDFLIEVNGVNVIKLGHKQVVSLIRQGGNRLLMKVVTVT
Da-shank3b 634 ALQYLESVDLEGVAWRAGLRTGDFLIEVNGVSVVKVGHRQVVSLIRQGGSRLVMKVVSVT
Da-shank3a 611 -----------------------FIIVIN-----------------QGGCW---------
consensus 721 .......................*.*..*.................***...........


Homo 666 RKPE-EDGARRRAPPPPKRAPSTTLTLRSKSMTAELEELASIRRRKGEKLDEMLAAA--A
Mus 665 RKPE-EDGARRRAPPPPKRAPSTTLTLRSKSMTAELEELASIRRRKGEKLDEILAVA--A
Bos 699 RKPE-EDGARRRAPPPPKRAPSTTLTLRSKSMTAELEELASIRRRKGEKLDEILAAA--A
Takifugu 683 RKPETEEVIRRKAPPPPKRAPSTTLTLRSKSMTAELEELASARRRRGERLDEMLAS---Q
Da-shank3b 694 RKPDTGDVVRKKAPPPPKRDPSTSLTLRSKSMTAELEELAS-RRRRGEKLDEMLSSPKEQ
Da-shank3a ------------------------------------------------------------
consensus 781 .... .................................................. . .


Homo 723 EPTLRPDIADADSRAATVKQRPTSRRITPAEISSLFERQGLPGP--------EKLPGSLR
Mus 722 EPTLRPDIADADSRAATVKQRPTSRRITPAEISSLFERQGLPGP--------EKLPGSLR
Bos 756 EPALRPDIADADSRAATVKQRPTSRRITPAEISSLFERQGLPGP--------EKLPGSLR
Takifugu 740 ESALRSQPSEADYRAATVKQRPTSRRITPAEISSLFERQGMTFHGGILHPGIERGHIPIP
Da-shank3b 753 VVVMRQRPVDSDSRAATVKQRPTSRRITQAEISSLLERQGLPIS--ELSLAVDKSHMQLP
Da-shank3a ------------------------------------------------------------
consensus 841 .. ......................................... ........


Homo 775 KGIPRTKSVGE---DEKLASLLEGRFPRSTSMQDPVREG-RGIPPPPQTAPPPPPAPYYF
Mus 774 KGIPRTKSVGE---DEKLASLLEGRFPRSTSMQDTVREG-RGIPPPPQTAPPPPPAPYYF
Bos 808 KGIPRTKSVGE---DEKLASLLEGRFPRSTSMQDSVREG-RGIPPPPQTAPPPPPAPYYF
Takifugu 800 KGMSRTKSFGATEEDRLSALADEHRFPRSSSMTDSLRDHPQTHPIPPPPQMAPPPPPYYH
Da-shank3b 811 RGMSRTKSFGN--DDRISALIGEHRFPRSSSMTDSFRQD--SIPPPPQTAPPPPPTPYFL
Da-shank3a ------------------------------------------------------------
consensus 901 ........... ......................... ....................

Homo 831 DSGPPPAFSPPPPPGRAYDTVRSSFKPGLEARLGAGAAGLYEPGAALGPLPYPERQKRAR
Mus 830 DSGPPPTFSPPPPPGRAYDTVRSSFKPGLEARLGAGAAGLYDPSTPLGPLPYPERQKRAR
Bos 864 DSGPPPAFSPPPPPGRAYDTVRSSFKPGLEARLGAGAPGLYDSGAALGPLPYPERQKRAR
Takifugu 860 DTGPPPGFCPPPPPSRT---QSQGHEPGVRSSFKPSSLDLSYEAAQRQAS-HIERQKKAR
Da-shank3b 867 DSGPPPSFLPPPPPSRA---ANQS-----RSSFRPG-----AEPKIHGPV-TTDRQRKTR
Da-shank3a ------------------------------------------------------------
consensus 961 ..................................... .... .. ..............


Homo 891 SMIILQDSAP---ESGDAPRPPPAATPPERPKR-RPRPPGPDSPYANLGAFSASLFAPSK
Mus 890 SMIILQDSAP---EVGDVPRPAPAATPPERPKR-RPRPSGPDSPYANLGAFSASLFAPSK
Bos 924 SMIILQDSAP---EAADASRPPPAATPPERPKR-RPRPPGPDSPYANLGAFSASLFAPSK
Takifugu 916 SMIILQDSSHLPVEPTEIPRPSAAT----PPERIKRKGRVIDNPYANVGQFSIGLYTPTK
Da-shank3b 913 SMIILQDTSHLPVEPTPITRPQTPTSGPVPPERGRRRGPPVENPYANVGRLS-AVYTPTK
Da-shank3a ------------------------------------------------------------
consensus 1021 .......... . ...... ........... ..........................


Homo 947 PQRRKSPLVKQLQVEDAQERAALAVGSPGPGGGSFAREPSPTHRGPRPGGLDYGAGDGPG
Mus 946 PQRRKSPLVKQLQVEDAQERAALAVGSPGPVGGSFAREPSPTHRGPRPGSLDYSSGEGLG
Bos 980 PQRRKSPLVKQLQVEDAQERAALAVGSPGPVVGSFAREPSPTHRGPRPSGLDYGPGDGPG
Takifugu 972 PQRKKSPLVKQLQVEDAQEKASLALAAAH------SRESSPSGRHPHTHGHTHTSRGKGP
Da-shank3b 972 PQRRKSPLVKQGPVEEG-------AAAHS------SRDPSPLGGS----RIPHSSRAEQF
Da-shank3a ------------------------------------------------------------
consensus 1081 .............................. ................ ......... .


Homo 1007 LAFGGP--GPAKDRRLEERRRSTVFLSVGAIEGSAPG-ADLPSLQPSRSIDERLLGTGPT
Mus 1006 LTFGGPSPGPVKERRLEERRRSTVFLSVGAIEGSPPS-ADLPSLQPSRSIDERLLGTGAT
Bos 1040 LAFGGP--APSKDRRLEERRRSTVFLSVGAIEGASPS-ADMPSLQPSRSIDERLLGPGAT
Takifugu 1026 FAAAIAGAVKDRERRLEERRKSTVFLSVGTMEGASTTSSDIPSLTQSHSIDERMLTRELG
Da-shank3b 1015 QQQVLS--ERARITPPGARRRPSVFLS---VEGGATEPQTTPLLSQSHSVDELAELPPPA
Da-shank3a ------------------------------------------------------------
consensus 1141 ...... .. ....................... .. .................. . .


Homo 1064 -AGRDLLLPSPVSALKPLVSGPSLGPSGSTFIHPLTGKPLDPSSPLALALAARERALASQ
Mus 1065 -TGRDLLLPSPVSALKPLVGGPSLGPSGSTFIHPLTGKPLDPSSPLALALAARERALASQ
Bos 1097 ITGRDLLLPSPVSALKPLVSGPSLGPSGSTFIHPLTGKPLDPSSPLALALAARERALASQ
Takifugu 1086 -------------QLPPPASALSPSPSGTTFIHPLTGKPLDPSSPLALALAARERALTSQ
Da-shank3b 1070 -------------PMLSPG----PPPGGTTFIHPLTGRPLDPSSPLALALAARERALSGR
Da-shank3a ------------------------------------------------------------
consensus 1201 ..........................................................


Homo 1123 APSRSPTPVHSPDADRPGPLFVDVQARDPERGSLASPAFSPR------------------
Mus 1124 TPSRSPTPVHSPDADRPGPLFVDVQTRDSERGPLASPAFSPR------------------
Bos 1157 APSRSPTPVHSPDTDRPGPLFVDVQARDSERGPLASPAFSPR------------------
Takifugu 1133 NQSPTSSPEPRTKPERISQGGIFIDLQTKESPQGEGVTTTPPFSPKSAKAMGHGVGAFGS
Da-shank3b 1113 N-TPTPTPSPTPSPTQG---------RAVERPETEGGATPP-------------------
Da-shank3a ------------------------------------------------------------
consensus 1261 ............ ........... .. ... .........


Homo 1165 -------------SPAWIPVPARREAEKVPREERKSPEDKKSMILSVLDTSLQRPAGLIV
Mus 1166 -------------SPAWIPVPARREAEKPPREERKSPEDKKSMILSVLDTSLQRPAGLIV
Bos 1199 -------------SPAWVPVPARREPEKVPREERKSPEDKKSMILSVLDTSLQRPAGLIV
Takifugu 1193 VLVPQPTKPQWTPSPSPLSFRQEMEAKVEERKEDKRLDDKKSMLISIVDTSQQKTAGLIM
Da-shank3b 1144 -----------------------------APLEAPPSNSWRDEPVSITETASQVTS----
Da-shank3a ------------------------------------------------------------
consensus 1321 ............... ...............................


Homo 1212 VHATSNGQEPSRLGGAEEER--PGTPELAPAPMQSAAVAEPLPSPRAQPPGGTPADAGPG
Mus 1213 VHATSNGQEPSRLG-AEEER--PGTPELAPAPMQAAAVAEPMPSPRAQPPGSIPADPGPG
Bos 1246 VHATSNGHEPSGLG-AEEQR--PGTPELAPAPTQSAVVAEPLPSPRAQPPGSAPTDPGPG
Takifugu 1253 VHATSNGQAEGLGSELDQARTSKVIEPSRSPSPRAKSPSPAVSQPQAATPSPQPAGPAQE
Da-shank3b 1171 -------GSPGSGRSLEEALAPPGVQNIQPALMDTEHTPPAVP---PTLPSPAPTLSNLT
Da-shank3a ------------------------------------------------------------
consensus 1381 ........... .. ... . .............. .............. .......

Homo 1270 QG---SSEEEPELVFAVNLPPAQLSSSDEETREELARIGLVPPPEEFANGVLLATPLAGP
Mus 1270 QG---SSEEEPELVFAVNLPPAQLSSSDEETREELARIGLVPPPEEFANGILLTTPPPGP
Bos 1303 QG---SSEEEPELVFAVNLPPAQLSSSDEETREELARIGLVPPPEEFANGVLLATPLPGP
Takifugu 1313 KSLAQGSSEEDVDQYTVTLPPAMLSSSDEETREELRKIGVVPPPDGFANGLLAQAQGPPP
Da-shank3b 1221 ARSMTMSSEEEAEPYTVTLPPALLSSSDEETREELRKIGLVPPPQPFANGLLIKETSKAT
Da-shank3a ------------------------------------------------------------
consensus 1441 .. ................................................ .. ...


Homo 1327 GPSPTTVPSPASGKPSSEPPPAP---------ESAADSGVEEADTRSSS----DPHLETT
Mus 1327 GPLPTTVPSPASGKPSSELPPAP---------ESAADSGVEEADTRSSS----DPHLETT
Bos 1360 GPSPTTVPGPASGKPSSEPPPAP---------ESAADSGVEEADTRSSS----DPHLETT
Takifugu 1373 QPQPPPPPPPPSAAAVSGKPSDPLEPPPVGESGSAADSGVEEADTRSSSERERDHHLETT
Da-shank3b 1281 LSISPSGSRPSIAKTSSGKASDS-----------TADSGVEDP------------HMETT
Da-shank3a ------------------------------------------------------------
consensus 1501 .. ..... ......... .... ................. .......


Homo 1374 STISTVSSMSTLSSESGELTDTHTS---FADGHTFLLEKPPVPPKPKLKSPLGKG-PVTF
Mus 1374 STISTVSSMSTLSSESGELTDTHTS---FADGHTFLLEKPPVPPKPKLKSPLGKG-PVTF
Bos 1407 STISTVSSMSTLSSESGELTDTHTS---FADGHTFLLEKPPVPPKPKLKSPLGKG-PVTF
Takifugu 1433 STVSTVSSMSTLSSECGEPADTHTTHTSYADGQTFVLDKPPVPPKPRLKSQIGGKGSVTF
Da-shank3b 1318 STVSTVSSMSTLSSESTDSAHAS-----------------------KPRCGVGRGRPAHL
Da-shank3a ------------------------------------------------------------
consensus 1561 ......................... ........................... ....


Homo 1430 RDPLLKQSSDSELMAQQHHAASAGLASAAG-------------PARPRYLFQRRSKLWGD
Mus 1430 RDPLLKQSSDSELMAQQHHAASTGLASAAG-------------PARPRYLFQRRSKLWGD
Bos 1463 RDPLLKQSSDSELMAQQHHAATAGLASAAG-------------PARPRYLFQRRSKLWGD
Takifugu 1493 RDPLLKQSSDSELLSQQQAAALAAAAGGAGLPSGGSASVTGLAPTKPRYLFQRRSKLWGD
Da-shank3b 1355 RDPLLKQSSDSELLPHPPSTG----------------------PSRPRYLFQRRSKLWGE
Da-shank3a ------------------------------------------------------------
consensus 1621 .............................. .................


Homo 1477 PVESRGLPGPEDDKPTVISELSSRLQQLNKDTRSLGEEPVGGLGSLLDPAKKSPIAAARL
Mus 1477 PVESRGLPGPEDDKPTVISELSSRLQQLNKDTRSLGEEPVGGLGSLLDPAKKSPIAAARL
Bos 1510 PVESRGLPGPEDDKPTVITELSSRLQQLNKDTRSLGEEPAGGLGGLLDPAKKSPIAAARL
Takifugu 1553 PVEPR-GPGVGLAKPSVMGELSSRLQQLNKDTRSLGEEP---LGASLDPGRKSPVAGARL
Da-shank3b 1393 EPRAQ-MGSSDESRPAAMG-----AELLSKDTHSLGEEPP--MGAPLDPGRRSPVGGARL
Da-shank3a ------------------------------------------------------------
consensus 1681 ....................................... ....................


Homo 1537 FSSLGELSSISAQ-RSPGGPGGGASYSVRPSGRYPVARRAPSPVKPASLERVEGLGAGAG
Mus 1537 FSSLGELSTISAQ-RSPGGPGGGASYSVRPSGRYPVARRAPSPVKPASLERVEGLGAGVG
Bos 1570 FSSLGELSTISAQQRSPGGPGGGASYPVRPGGRYPVARRAPSPVKPASLERVEGLGAGAG
Takifugu 1609 FSSLGELHTISQR-------SYGTTFTIRPGSRYPVTRRTQSPGSGSPDRGDPLGRFTSF
Da-shank3b 1445 FSSLGELHTISQR-------SYGTTFTIRPGSRYPVTRRTPSPG-ATPERSEPLGPVRTF
Da-shank3a ------------------------------------------------------------
consensus 1741 ............. ............................................ .


Homo 1596 GAGRPFGLTPPTILKSSSLSIPHEPKEVRFVVRSVSARSRSPSPSPLPSPASGPGPGAPG
Mus 1596 GAGRPFGLTPPTILKSSSLSIPHEPKEVRFVVRSVSARSRSPSPSPLPSPSPGSGP-SAG
Bos 1630 GAGRPFGLTPPTILKSSSLSIPHEPKEVRFVVRSVSARSRSPSLSPLPSPAPGPGPGAPS
Takifugu 1662 GLPTSPTTPPQTILKSSSLSLPQEPKEVRFVMRSSSARSRSRSPSPSPSHSPR----LGS
Da-shank3b 1497 G----PHHHHHTILKSSSLSLPQEPKEVRFVMRSASARARSRSPSPSPCASPCPSPVLGA
Da-shank3a ------------------------------------------------------------
consensus 1801 ........................................................ ..


Homo 1656 PR-----RPFQQKPLQLWSKFDVGDWLESIHLGEHRDRFEDHEIEGAHLPALTKDDFVELGV
Mus 1655 PR-----RPFQQKPLQLWSKFDVGDWLESIHLGEHRDRFEDHEIEGAHLPALTKEDFVELGV
Bos 1690 PR-----RPFQQKPLQLWSKFDVGDWLESIHLGEHRDRFEDHEIEGAHLPALTKDDFVELGV
Takifugu 1718 PLLTLRPFHQKPLHLWNKYDVGDWLESINLGEHRAGFQEHEIEGSHLPALTKDDFAELGV
Da-shank3b 1553 PLLALRPFRQRPLALWSKYDVGEWLESVGLGEHRARFLEHEIEGAHLPALTKDDLAELGV
Da-shank3a ------------------------------------------------------------
consensus 1861 .. .......................................................

Homo 1713 TRVGHRMNIERALRQLDGS
Mus 1712 TRVGHRMNIERALRQLDGS
Bos 1747 TRVGHRMNIERALRQLDGS
Takifugu 1778 TRVGHRMNIERALKQLLES
Da-shank3b 1613 TRVGHRMNIERALKQLLES
Da-shank3a -------------------
consensus 1921 ...................

**Supplementary Material, Fig. S2** Protein alignment and domain annotation analysis of *SHANK3* orthologs contained *Homo sapiens, Mus musculus, Bos Taurus, Takifugu rubripes* and *Danio rerio-*shank3b and shank3a. The major functional domains that define the *SHANK3* gene family are highlighted: ANK (green), SH3 (light blue), PDZ (purple), and SAM (yellow).


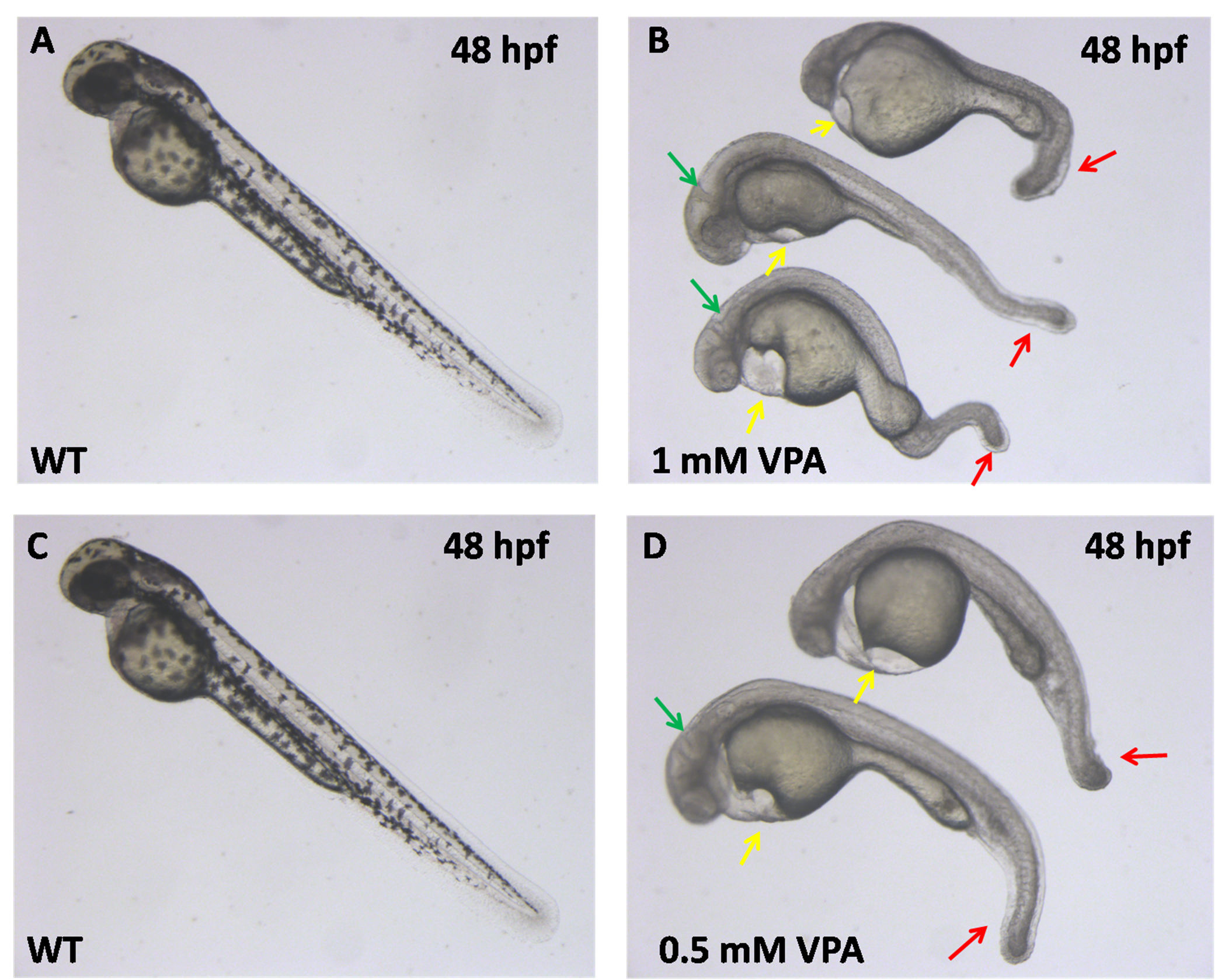


**Supplementary Material, Fig. S3**

(A and B) Most embryos exhibited severe phenotypic abnormalities when treated with 1mM VPA, such as [developmental](http://dict.youdao.com/w/developmental/) [retardation](http://dict.youdao.com/w/retardation/) (100%, 70/70), reduced pigmentation (100%，70/70, green arrow), spinal curvature (43%, 30/70, red arrow) and pericardial effusion (64%, 45/70, yellow arrow).

(C and D) Embryos that exposed to 0.5 mM VPA displayed relatively fewer defects, including [developmental](http://dict.youdao.com/w/developmental/) [retardation](http://dict.youdao.com/w/retardation/) (100%, 70/70), reduced pigmentation (83%，58/70, green arrow), spinal curvature (20%, 14/70, red arrow) and pericardial effusion (40%, 28/70, yellow arrow).


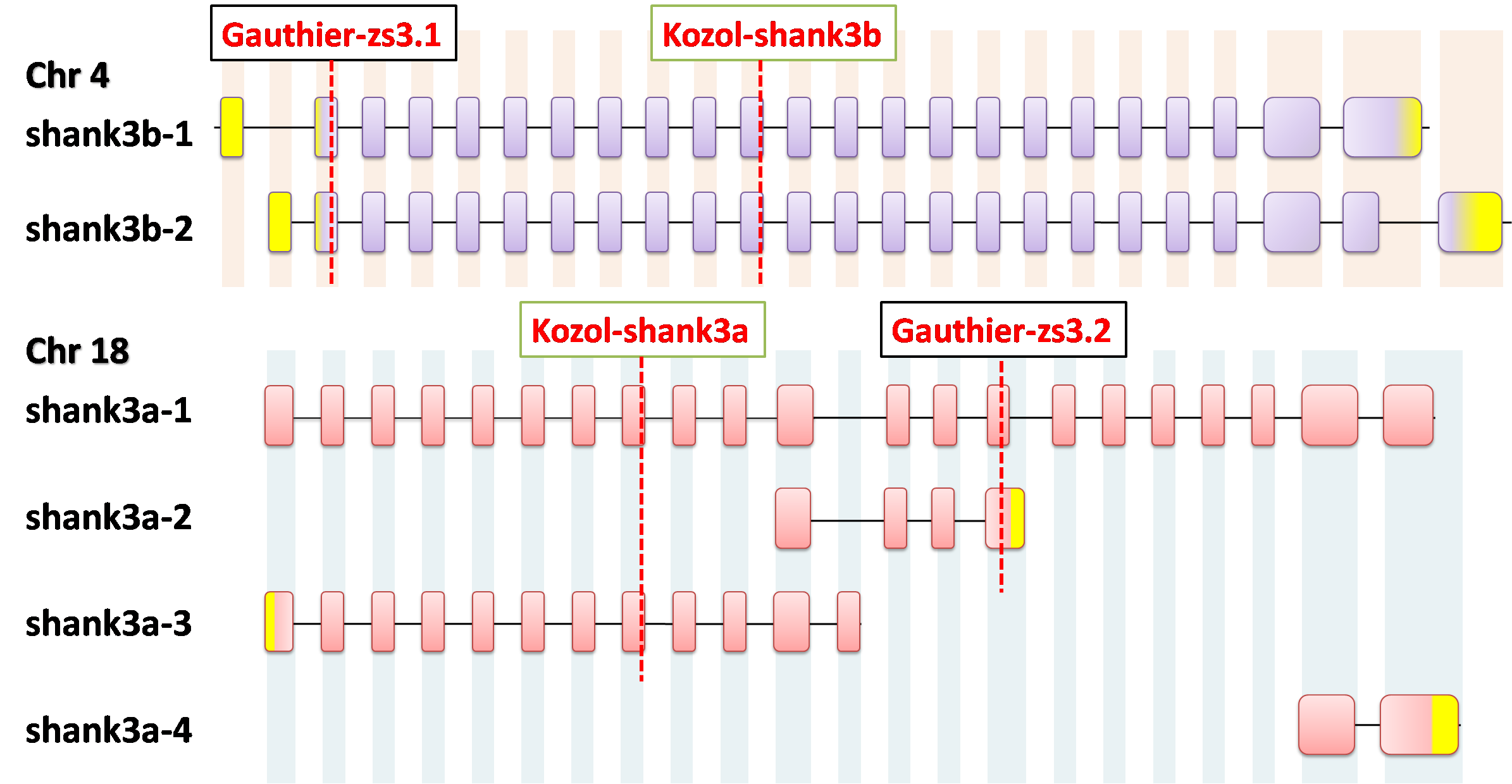


**Supplementary Material, Fig. S4** Upon performing a BLAST search of the morpholino oligonucleotides (red dotted line) used in the studies of Kozol et al and Gauthier et al, we found that Kozol-shank3a corresponded to *shank3a-1* and *shank3a-3* in our study, and Kozol-shank3b corresponded to *shank3b-1* and *shank3b-2*. In addition, Gauthier-zs3.1 was similar to *shank3b-1* and *shank3b-2*; Gauthier-zs3.2 and *shank3a-1* and *shank3a-2* were similar.

**Supplementary Material, Table S1.** Transcript-specific oligonucleotide primers used for q-PCR in this study.

__________________________________________________________________________________________________

**Shank3 transcripts Oligonucleotide (5’- 3’) Amplicon Ensemble**

**length (bp) transcript ID**

**__________________________________________________________________________________________________**

*Shank3b-1* GGTTTTATTAGGGTTGTGAGGCCG 191 ENSDART00000145613

CCCAGGAGAGGTCCGAATACTGTC

*Shank3b-2* TCCAGGTCGAAGGGTTGTGAGG 191 ENSDART00000091809

CCAGGAGAGGTCCGAATACTGTCAT

*Shank3a-1* TCACCCTGAGGTCCAAAAGCATG 200 ENSDART00000139505/

CTTGTCGCTCAAAAAGAGAACTGATTTC our study

*Shank3a-2* CTCGACTTGAGACGAGAGAGGATCG 176 ENSDART00000139188

CATCTTACCTTTTGCTCCTCGCAAG

*Shank3a-3* CCTCAGCCCGGGGACGGT 197 ENSDART00000091805

TTAATTCTTTGACCCTCTCTCCGCG

*Shank3a-4* TCGGTCCAAGACTCTTCAGCAGTTT 580 ENSDART00000092456

AGGCAGAGGGAGGCGCGT

*β-actin*  CGAGCTGTCTTCCCATCCA 102

TCACCAACGTAGCTGTCTTTCTG

**___________________________________________________________________________________________________**

**Supplementary Material, Table S2.** Gene-specific oligonucleotide primers for in situ hybridization RNA probe synthesis.

__________________________________________________________________________________________________

**Gene Oligonucleotide (5’- 3’) Amplicon Ensemble**

**length (bp) transcript ID**

**__________________________________________________________________________________________________**

*Shank3b-1* gagctgaaatgcaggatgtagtgcc 481 ENSDART00000145613

gaaatgcacacaaatccagatggtg

*Shank3a-1* tcaactccgcctgagagaat 360 ENSDART00000139505/

gtccttcacagcatcagcaa our study

**___________________________________________________________________________________________________**
